# Abstracts and Selections

**Published:** 1905-02

**Authors:** 


### Abstracts and Selections.

"In the Japanese army the medical officer is omnipresent. You will find him in countless places where in an American or British army he has no place. He is as much at the front as in the rear. He is with the first screen of scouts with his microscope and chemicals, testing and labeling wells so that the army to follow shall drink no contaminated water. When the scouts reach a town, he immediately institutes a thorough examination of its sanitary condition, and if contagion or infection is found he quarantines and places a guard around the dangerous district. Notices are posted so that the approaching column is warned and no soldiers are billeted where danger exists. Microscopic blood tests are made in all fever cases—and bacteriological experts, fully equipped, form part of the staff of every divisional headquarters.

"The medical officer also accompanies foraging parties, and with the commissariat officers samples the various food, fruits, and vegetables sold by the natives along the line of march long before the arrival of the army. If the food is tainted or the fruit overripe, or the water requires boiling, notice is posted to that effect, and such is the respect and discipline of every soldier from commanding officer to the file in the ranks that obedience to its order is absolute.

"The medical officer is also found in camp, lecturing the men on sanitation and the hundred and one details of personal hygiene, how to cook—to eat—and when not to drink—to bathe and even to the direction of the paring and cleansing of the finger nails to prevent danger from bacteria. Long before the outbreak of hostilities he was with the advance agents of the army, testing provisions that were being collected for troops that were to follow, and as a consequence of these precautions, he is not now found treating thousands of cases of intestinal diseases, diarrheas or dysenteries, contagion, and fevers that follow improper subsistence and neglected sanitation—diseases that have brought more campaigns to disastrous terminations than the strategies of opposing generals or the bullets of their followers. * * *

"From the standpoint of a humanitarian and a lover of his kind, I tell you it was a positive delight to visit that great series of hospitals, from Tokyo to Sasebo, with their long wards filled to overflowing with wounded, suffering soldiers—the legitimate victims of war, their faces full of health and hope, despite their fearful wounds in the long, hard campaign of five or six months in Manchuria, their chief desire to know how soon they could rejoin their comrades, and to contrast them, in memory, with the vivid picture of the poor, wan, emaciated, and almost hopeless faces that crowded the wards of our hospitals in Cuba, and Porto Rico, in Tampa, Chattanooga, and Camp Alger, and Montauk Point in 1898—and in the Philippines in 1899 and 1900—the innocent, unwounded, and illegitimate victims of another conflict, which, in comparison with the one now waging, would be considered no more than a skirmish among outposts.

"*If wars are inevitable, and the slaughter of men must go on* (and I believe wars *are* inevitable, and that most of them are ultimately beneficial), then, for the love of God, let our men be killed legitimately on the field, fighting for the stake at issue—not drop them by the wayside by preventable diseases as we did in the Spanish-American war—1400 for every 100 that died in action. It is for the 1400 poor devils who are sacrificed—never for the 100 who fall gallantly fighting—that I offer my prayer.

"And yet, should occasion arise for the gathering of another army of 250,000 next summer, what evidence is submitted to prove that the lamentable scenes of 1898, with all their nauseating details, would not be repeated? Where, as in Porto Rico, Tampa, and Chattanooga, no fighting was done, but where more sick and invalided were gathered at one time than would overload any dozen transports and hospital ships with men who never smelled powder or saw a hardship of real war, and who, had they been properly subsisted on the principle of the Japanese today, would have returned to their homes and vocations, healthy and happy as after a summer's outing? I ask what tangible evidence is submitted to show that history would not repeat itself, and that such an army, gathered hastily, would not again be brought almost to its knees, through the same ignorance and incomptency. We have recently heard much of the reorganization of the American army and the creation of a general staff. Commanding that staff is an officer as courageous, as gallant, as heroic and, I believe, as representative, as ever drew a sword, and yet the importance of this momentous subject, the study of preventable disease, and the saving of eighty men out of every 100 that always die in war is considered of such minor import that no place is found on it for a medical representative.

"The three great lessons to be learned from the Japanese war [are] from the medical, the commissariat, and transport departments. The Japanese authorities permitted our government to send five military attaches to accompany their army in the field. Was a surgeon or a quartermaster or a commissary officer detailed? No. They represented the life-saving and life-preserving departments and were omitted. The killing departments got the appointments—the cavalry, ordnance, infantry, etc.—and today Japanese officers are laughing in their sleeves at our senseless failure to have representatives on what they consider their three vital points, while the only weak feature of their army, its cavalry, is considered worthy of special study. Certainly 'it is to laugh.' But what can be expected of a government that after its terrible lessons of 1898-1899 still insists—especially in the tropics—of subsisting its army on a ration so rich and elastic (lovely term, that elastic), so elastic that when in the emergency of war its elasticity is tested, it bursts its bands, and is found to consist of pork and beans and fermenting canned rubbish that in six weeks prostrates 50 per cent of its 250,000 units with intestinal diseases, and sends 3000 to their last homes, to say nothing of the enormous number of invalided and the 75,000 pension claims? That in its famous reorganization fails utterly to recognize one of the most important of all the departments, namely, that of sanitation, as it is recognized by the Japanese today? That holds its great life-preserving department in such light esteem, that but one officer in the entire army can even reach the rank and emoluments of a brigadier-general? That on its general staff fails to have a single representative of this department, and if any, only a young, inexperienced man of inferior rank, instead of the ablest and most experienced officer in or out of the service, one of international reputation like our retired Surgeon-General Sternberg, whose rank should not be less than that of a majorgeneral, and whose opinions would carry weight in councils of war? * * *

"Why, I ask again, should we expect reforms from authorities who, in their great preparatory schools, West Point and Annapolis, furnish the cadets no instruction in the important studies of physiology and hygiene, so that when they come to command the fighting units of the army they can be prepared to guard them against the silent foe which scores 80 per cent of the deaths! Like the rest of the world, we go blundering on, spending millions annually for the maintenance of these great military schools and arsenals and war colleges, educating men in the art of human destruction, while the more formidable adversary in the ranks—the grim specter that kills 80 per cent—is left comparatively unheeded!—*From Maj. Seaman's paper, Surgeon U. S. A., quoted in Literary Digest.*

